# Split scar sign to predict complete response in rectal cancer after neoadjuvant chemoradiotherapy: systematic review and meta-analysis

**DOI:** 10.1007/s00330-023-10447-z

**Published:** 2023-11-18

**Authors:** Giovanni Brondani Torri, Camila Piovesan Wiethan, Felipe Welter Langer, Guilherme Strieder de Oliveira, Alice Villa Bella Meirelles, Natally Horvat, Justin Ruey Tse, Adriano Basso Dias, Stephan Altmayer

**Affiliations:** 1 Department of Radiology and Diagnostic Imaging, University Hospital of Santa Maria, Federal University of Santa Maria, Santa Maria, Rio Grande Do Sul 97105-900 Brazil; 2 Clinics Hospital of Porto Alegre, R. Ramiro Barcelos, Porto Alegre, 235090035903 Brazil; 3 Clinics Hospital, Federal University of Minas Gerais, Av. Prof. Alfredo Balena, 110 - Santa Efigênia, Belo Horizonte, 30130-100 Brazil; 4 Department of Radiology, Memorial Sloan Kettering Cancer Center, 1275 York Ave, New York, NY 10065 USA; 5 Department of Radiology, Stanford University School of Medicine, 300 Pasteur Drive, Stanford, CA 94305-5105 USA; 6 Department of Medical Imaging, University of Toronto, 263 McCaul Street, 4Th Floor, Toronto, ON M5T 1W7 Canada

**Keywords:** Magnetic resonance imaging, Rectal neoplasms, Watchful waiting, Split scar sign

## Abstract

**Objectives:**

Magnetic resonance imaging (MRI) is the modality of choice for rectal cancer initial staging and restaging after neoadjuvant chemoradiation. Our objective was to perform a meta-analysis of the diagnostic performance of the split scar sign (SSS) on rectal MRI in predicting complete response after neoadjuvant therapy.

**Methods:**

MEDLINE, EMBASE, and Cochrane databases were searched for relevant published studies through June 2023. Primary studies met eligibility criteria if they evaluated the diagnostic performance of the SSS to predict complete response on pathology or clinical follow-up in patients undergoing neoadjuvant chemoradiation. A meta-analysis with a random-effects model was used to estimate pooled sensitivity and specificity, area under the curve (AUC), and diagnostic odds ratio (DOR) of the SSS.

**Results:**

A total of 4 studies comprising 377 patients met the inclusion criteria. The prevalence of complete response in the studies was 21.7–52.5%. The pooled sensitivity and specificity of the SSS to predict complete response were 62.0% (95% CI, 43.5–78.5%) and 91.9% (95% CI, 78.9–97.2%), respectively. The estimated AUC for SSS was 0.83 (95% CI, 0.56–0.94) with a DOR of 18.8 (95% CI, 3.65–96.5).

**Conclusion:**

The presence of SSS on rectal MRI demonstrated high specificity for complete response in patients with rectal cancer after neoadjuvant chemoradiation. This imaging pattern can be a valuable tool to identify potential candidates for organ-sparing treatment and surveillance.

**Clinical relevance statement:**

SSS presents high specificity for complete response post-neoadjuvant. This MRI finding enhances rectal cancer treatment assessment and aids clinicians and patients in choosing watch-and-wait over immediate surgery, which can potentially reduce costs and associated morbidity.

**Key Points:**

*•Fifteen to 50% of rectal cancer patients achieve complete response after neoadjuvant chemoradiation and may be eligible for a watch-and-wait strategy.*

*•The split scar sign has high specificity for a complete response.*

*•This imaging finding is valuable to select candidates for organ-sparing management.*

**Supplementary information:**

The online version contains supplementary material available at 10.1007/s00330-023-10447-z.

## Introduction

Colorectal cancer is the third most common malignancy in the world with a third of cases localized to the rectum [1]. By 2030, its incidence is expected to increase to up to 2.2 million new cases and more than 1 million deaths yearly [1]. Current therapeutic strategies for rectal cancer are guided by initial staging, which depends upon local extension assessed by magnetic resonance imaging (MRI) [2]. Standard treatment of locally advanced rectal cancer (LARC) involves neoadjuvant chemoradiotherapy (CRT) followed by surgical resection through total mesorectal excision (TME) [3]. However, TME carries significant postoperative morbidity that affects quality of life, with approximately 12% of patients experiencing complications; of those, 3.8% require surgical, endoscopic, or radiological intervention [4].

Studies indicate that up to 15–50% of patients achieve pathological complete response (pCR) without surgery [1, 3, 5, 6]. However, conventional management strategies identify these patients only postoperatively. Ideally, better restaging techniques would identify complete responders who could benefit from a “watch-and-wait” (WW) strategy instead of surgery, reducing treatment costs and postoperative care-related morbidity without compromising long-term mortality [7, 8].

**Fig. 1:**
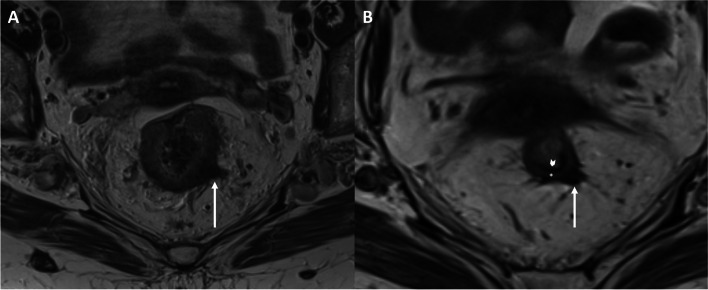
Split scar sign. **A** Axial T2-weighted image without fat-suppression depicting the baseline T stage, with a locally advanced rectal cancer that extends to the mesorectum at 4 o’clock (arrow). **B** Axial T2-weighted image without fat-suppression of the same patient after neoadjuvant therapy depicts the split scar sign, consisting of an inner hypointense mucosa (arrowhead), an intermediate signal *muscularis propria* (asterisk), and in this case, an outer hypointense layer of perirectal fibrosis (arrow)

The “split scar sign” (SSS) in restaging rectal MRI is a promising tool to identify potential candidates for a WW strategy rather than immediate surgery [3]. The sign consists of a regular hypointense scar on T2-weighted imaging (T2WI), indicating fibrosis in the submucosa, with an underlying layer of intermediate signal intensity at the *muscularis propria* and a third outermost hypointense layer corresponding to perirectal fibrosis (Fig. 1). A disruption of the hypointense scar by intermediate T2WI signal would indicate a residual or recurrent tumor. While the initial study reporting the SSS showed a specificity of 97% in identifying complete responders, subsequent studies have demonstrated variable diagnostic performance [3, 9–11].

The goal of our systematic review and meta-analysis was to determine the diagnostic value of SSS in predicting complete response in patients who underwent neoadjuvant CRT for rectal cancer.

## Methods

### Search strategy and inclusion criteria

This systematic review and meta-analysis was performed following the established recommendations outlined in the Preferred Reporting Items for Systematic Reviews and Meta-Analyses (PRISMA) guidelines [12], and the Cochrane Handbook for Systematic Reviews of Diagnostic Test Accuracy [13]. This study was registered in the International Prospective Register of Systematic Reviews (PROSPERO) under the number CRD42023438309.

All relevant publications in the MEDLINE, EMBASE, and Cochrane Library were searched from January 2020 to June 2023, as the first report of the SSS was in 2020 [3]. Additionally, a snowballing search was performed in the references section of the studies initially included to identify additional eligible studies. Studies were included if they evaluated the diagnostic performance of the SSS to identify patients with either rectal cancer complete response (considered pCR or clinical complete response during follow-up). We excluded studies if they (1) were not published in English; (2) were conference abstracts or unrefereed preprints; and (3) had fewer than 10 patients. The terms used in the database search are included in Appendix E1.

Based on the PICOS (population, intervention, comparator, outcome, study design) approach, we defined our target population as patients submitted to neoadjuvant therapy for colorectal cancer; “intervention” as SSS; outcome as diagnostic accuracy of this sign to define a complete response; and study design as controlled or comparative, randomized or nonrandomized studies, and prospective or retrospective observational studies [14].

### Study selection

Two reviewers, board-certified radiologists with 5 years of post-graduate experience in rectal MRI, conducted separate evaluations of the initial search based on identifying studies that met the eligibility based on their titles and abstracts. Subsequently, the selected studies underwent full-text assessment by the same reviewers, who then made the final selection of articles that aligned with the inclusion and exclusion criteria. Any disagreement between the two reviewers was decided by a third reviewer.

### Data extraction

The two reviewers who conducted the study selection independently extracted data from the selected studies into a standardized form including (a) study characteristics: authors, year of publication, study design, sample size, number of image reviewers, reader time experience; imaging technique (technical specifications of MRI machine, imaging protocol, use of enema); reference standard for complete clinical response or pathological complete response; (b) demographic characteristics: mean age, gender; cancer initial staging; distance from anal verge; mean interval between radiotherapy and imaging; proportion of complete response, near-complete response, and partial response; and (c) diagnostic performance of MRI, including the number of true-positives (TP), false-positives (FP), false-negatives (FN), true-negatives (TN), sensitivity, and specificity. For studies in which more than one reader evaluated the same patient, the numbers for the contingency table were acquired in the following order: consensus reading, then average performance among readers. Any disagreement between the two reviewers was resolved in consensus with a third reviewer.

### Quality assessment

To evaluate the potential for bias, each study included in the analysis was assessed independently by two reviewers using the Quality Assessment of Diagnostic Accuracy Studies (QUADAS-2) framework [15]. This tool comprises four main domains: patient selection (D1), index test (D2), reference standard (D3), and flow and timing (D4). These domains are subsequently evaluated for potential bias and rated for applicability, categorized as “high,” “low,” or “unclear.” Each study was further classified into a binary category: “low risk of bias” if it received a “low” rating across all bias and applicability domains, or “at risk of bias” if one or more domains were deemed “high risk of bias” or “unclear.” In case of any discrepancies between the two reviewers, the same third reviewer was involved to resolve in consensus.

### Statistical analysis

The pooled diagnostic sensitivity and specificity were obtained using a random-effects model. The Clopper-Pearson method was used to compute the 95% confidence intervals [16]. A summary receiver-operating characteristic curve (SROC) for the diagnostic performance of the SSS was obtained and the area under the curve (AUC) was estimated using parametric bootstrapping [17, 18]. Additionally, a natural logarithm transformation was applied to the diagnostic odds ratio (DOR), which serves as a summary indicator of test performance [19]. The heterogeneity of studies was qualitatively and quantitatively assessed and an *I*^2^ > 50% was considered substantial heterogeneity. The correlation between logit sensitivity and 1—specificity was calculated to assess the potential for a threshold effect, and a coefficient (*ρ*) ≥ 0.6 was interpreted as significant [20]. Data analysis was conducted using RStudio version 2023.06.0 + 421.

## Results

### Study selection and description

**Fig. 2:**
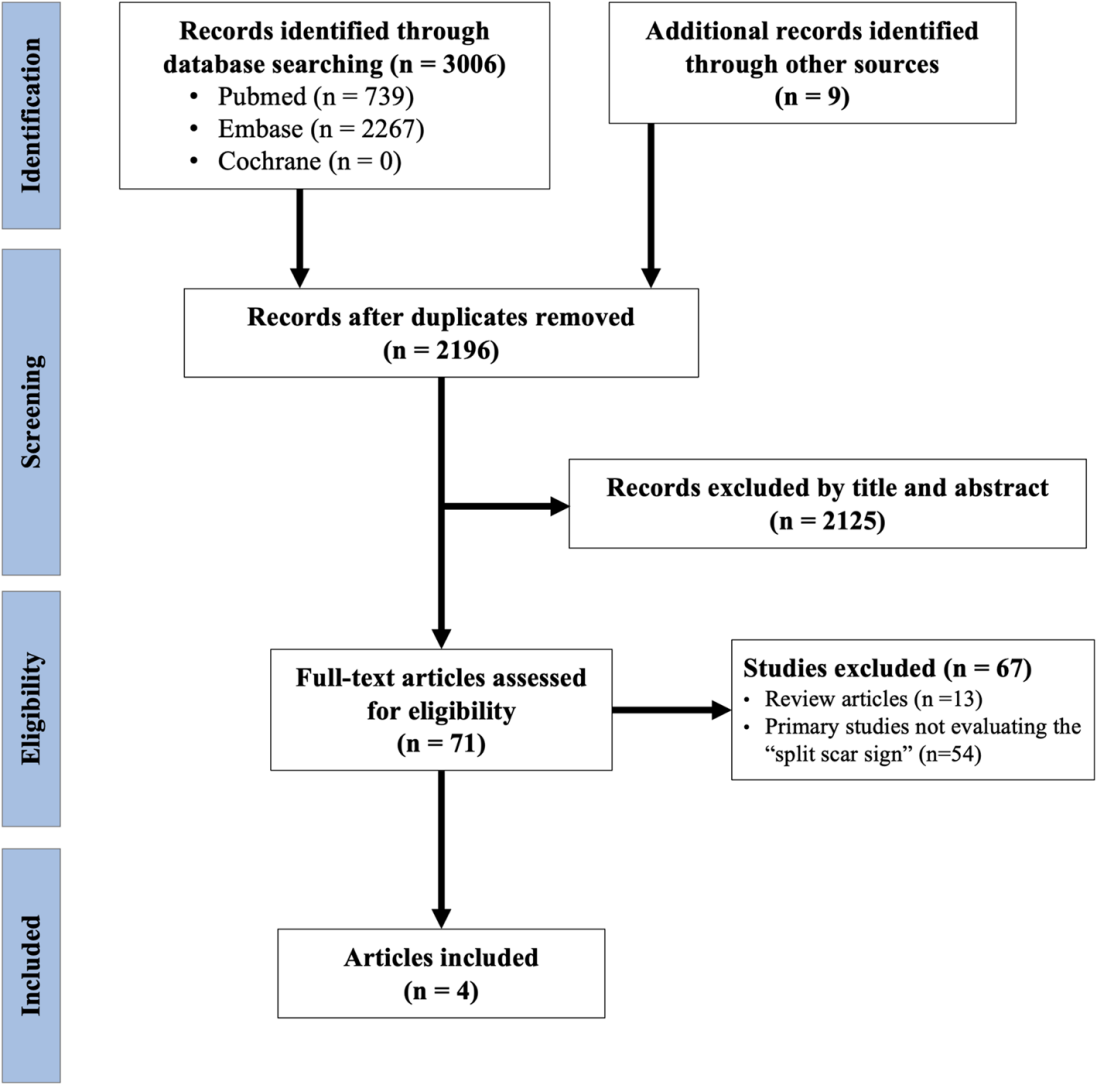
PRISMA flow chart

The initial search yielded 3015 articles (Fig. 2), from which 71 articles were reviewed, and 4 articles were considered eligible [3, 9–11]. Three of the studies were retrospective; of these, two were single-center and one was multi-center. The remaining study was a single-center and prospective (Table 1). Three studies used SSS as defined in its original description (solely on T2WI), while one article used a modified SSS, which combined T2WI and diffusion-weighted images (DWI). The included studies in this analysis had sample sizes ranging from 40 to 189 patients, with a total of 377 patients across all studies.

**Table 1:** Studies characteristics

Study	Year	Country	Design	Sample	Readers	Follow-up	Standard reference	Complete response*	Surgical intervention
Santiago et al	2020	Portugal	R	58	2	12–62 months	pCR or clinical response > 12 months	43%	59%
Popita et al	2022	Romania	P	40	2	6–24 months	pCR or clinical response > 6 months	52.5%	70%
El Khalabi et al	2022	Netherlands (10 centers)	R	90	22	> 24 months	pCR or clinical response > 24 months	30%	93%
Yuan et al	2023	China	R	189	3	3–27 months	pCR	21.7%	100%

*R*, retrospective; *P*, prospective; *pCR*, pathological complete response

^*^As defined in the standard reference column

The reference standard to determine complete response was a combination of pCR and complete clinical response on follow-up in three studies, while Yuan et al relied exclusively on pCR. The minimal follow-up interval to consider a complete clinical response differed among studies, and time endpoints of 6 months, 1 year, and 2 years were used (Table 1). In the three studies that considered complete clinical response as an outcome, a mean of 11% (range 7–41%) of patients were considered complete responders, based on clinical, endoscopic, and imaging assessment. The remaining patients underwent pathological evaluation of the resected specimens. Reassessment intervals differed slightly among studies, with the following approximate timeframes: every 3 months in the first year, every 4–6 months during the second year, and every 6–12 months thereafter.

All studies had more than one MRI reader and three of those described individually each reader’s diagnostic performance. The study by El Khababi et al demonstrated a low interobserver agreement (IOA) of 0.17 (individual reader’s IOA used quadratic kappa weighting, while the group agreement described was calculated using Krippendorff’s alpha). The remaining studies had a range of Cohen’s kappa of 0.56–0.80 (Cohen’s kappa was employed for individual IOA and Yuan et al calculated the consistency among readers with the Kendall coefficient of concordance).

MRI systems and protocols differed among studies. Some datasets were acquired on 1.5 T and one dataset was acquired solely on 3.0 T. Axial T2WI slice thickness was > 3 mm at least in part of two studies. High *b*-value on DWI was ≥ 800 in three of the studies. Three of the studies employed rectal enema, while the remaining was not explicit about this information. One of the studies reported the use of spasmolytics. Further details of the studies, including MRI protocols, are presented in Table E1.

### Quality of the studies

The results of the QUADAS-2 assessment are presented in Figure E1. The four studies were considered to have a low risk of bias in participant selection. All studies reported MRI readers’ blinding to the reference standard and outcome, although one of the studies was single-centered and retrospective, in which it is not possible to exclude memory bias.

### Overall diagnostic accuracy

**Fig. 3:**
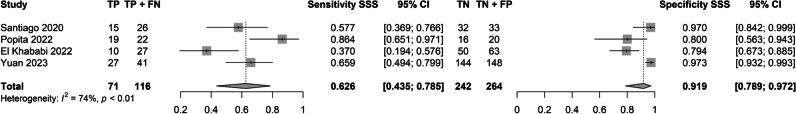
Forest plot of the primary analysis

**Fig. 4:**
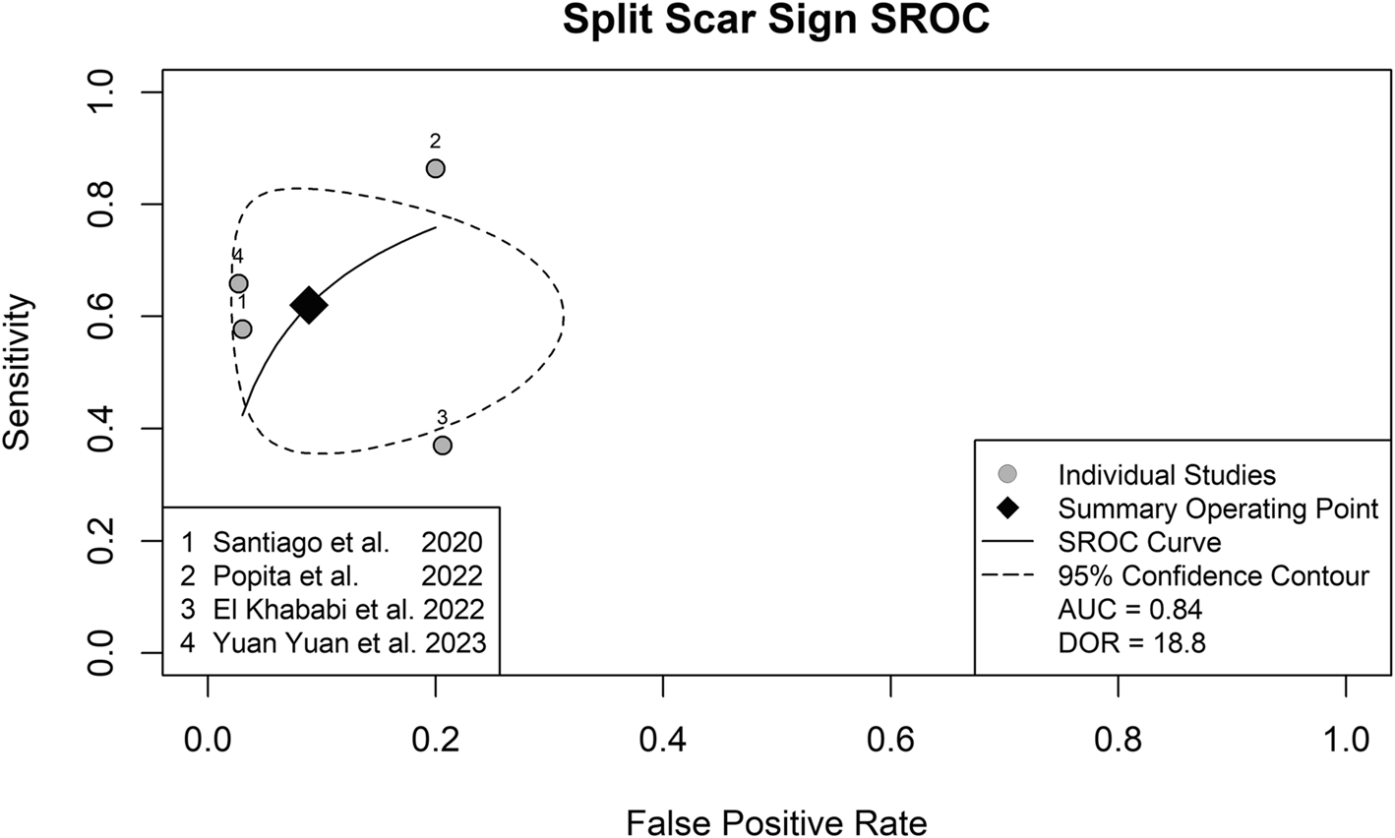
Summary receiver-operating characteristic curve for the primary analysis

The pooled analysis of studies that employed the SSS is summarized in Fig. 3. The combined sensitivity and specificity of SSS were respectively 62% (95% CI, 43.5–78.5%) and 91.9% (95% CI, 78.9–97.2%). The SROC is shown in Fig. 4 with an estimated AUC of 0.83 (95% CI, 0.56–0.94). The DOR was 18.8 (95% CI, 3.65–96.5). There was high heterogeneity for both sensitivity (*I*^2^ = 74%) and specificity (*I*^2^ = 83%), without substantial threshold effect to account for the heterogeneity (*ρ* = 0.08).

### Sensitivity analysis

A secondary analysis was performed to explain potential sources of heterogeneity among the studies. The study by El Khababi et al demonstrated the lowest sensitivity and an outlier position in the SROC curve (Fig. 4). Sensitivity and specificity obtained without El Khababi et al were 69.4% (95% CI, 55.4–80.5%; heterogeneity *I*^2^ = 54%) and 94.7% (95% CI, 84.1–98.4%; *I*^2^ = 78%), respectively (Supplementary Figures E2 and E3). The correlation between the logit transformation of sensitivity and specificity was almost perfect (*ρ* = 0.96) in the sensitivity analysis.

## Discussion

In this systematic review and meta-analysis comprising 4 studies with 377 patients, the SSS on MRI demonstrated a specificity of > 90% to predict complete response after neoadjuvant CRT for rectal cancer. Although the sign has modest sensitivity, when present, it may assist clinicians and patients opting for a “watch-and-wait” (WW) strategy instead of immediate surgical management, potentially reducing costs and the morbidity related to the surgery. Considering an average proportion of complete responders of 38% in this sample and the diagnostic performance in our meta-analysis, the post-test probability (positive predictive value) of complete response in the presence of SSS would be around 83%. Moreover, considering the Organ Preservation for Rectal Adenocarcinoma (OPRA) trial results, in which up to half of patients presented complete response after neoadjuvant therapy, even more optimistic outcomes could be expected [6].

After the introduction of WW strategies in 2004, rectal MRI became the primary technique to select eligible patients for this strategy [5]. WW has less morbidity and better functional outcomes than TME, with comparable survival in 5 years and a rate of distant metastasis, but at the cost of increased local recurrence [21]. In patients with complete clinical response, recurrence-free survival is about 94%, and distant metastasis survival is about 84% [6]. Although recurrence may occur, salvage therapy is feasible for almost all patients who relapse [22].

During the WW follow-up, MRI is recommended every 6 months for at least 3 years [23]. The most studied response evaluation method on rectal MRI during follow-up is the treatment regression grade (mrTRG) classification. This classification system, derived from the pathological tumor regression grading system, evaluates treatment-induced fibrosis on post-neoadjuvant chemoradiotherapy MRI scans [24]. The mrTRG is classified from 1 to 5, with a mrTRG scale of 1 showing a sensitivity and specificity of 32.3% and 93.5% respectively for pCR in a recent meta-analysis of 916 patients [24, 25]. On the other hand, when considering the presence of either a mrTRG 1 or 2, the sensitivity was 69.9% and specificity 62.2% [25]. Thus, SSS has shown higher specificity and comparable sensitivity in relation to mrTRG 1 to predict complete response, although the outcomes of the studies were based on different grounds (pCR vs. a combination of pCR and complete clinical response during follow-up). Nonetheless, both SSS and mrTRG refer only to the treatment response at the primary tumor site, while eligibility for a WW strategy should be individualized and considered on the basis of other factors, such as persistence of suspicious nodes or extramural venous invasion, among others.

The results of this study have implications for rectal cancer management. Given its high specificity, patients with a positive sign could be eligible for a WW strategy. As the SSS has shown only a modest sensitivity, a negative sign does not exclude the possibility of a complete response. Perhaps, patients could be optimally evaluated by using simultaneously the SSS and mrTRG classification, in which patients with mrTRG1 could also be complete responders with high specificity. With this approach, an increase in the number of suitable patients for a WW strategy would be expected, with the benefits of lower treatment costs and lower morbidity, and comparable rates of survival. Yuan et al employed a modified SSS, which added DWI acquisition to the evaluation [10]. In this study, the authors used DWI in a binary assessment of the presence or absence of high signal intensity on high *b*-value diffusion-weighted images, with apparent diffusion coefficient maps to confirm true diffusion restriction. Their study found that all readers consistently had higher sensitivity with lower specificity with DWI alone, while T2WI alone resulted in lower sensitivity but higher specificity. The modified SSS had an overall increased accuracy in relation to T2WI. Moreover, a meta-analysis with 8 studies suggested that the addition of DWI can significantly enhance diagnostic performance in other response evaluation methods that rely on T2WI [26]. Because a typical restaging MRI includes at least a T2WI and DWI, we believe the modified SSS had to be included in our study and should be further investigated as an alternative to the originally proposed SSS in future studies.

Imaging techniques for rectal staging are critical to ensure quality and inter-study reproducibility during follow-up. The most recent contributions in this regard are the recommendations by the European Society of Gastrointestinal Abdominal Radiology (ESGAR) and the Society of Abdominal Radiology (SAR). The 2016 ESGAR consensus meeting and 2017 SAR recommendations bring the latest recommendations in this regard [27, 28]. Both consensuses emphasize the necessity of a high-resolution T2WI, with an axial slice thickness optimally ≤ 3 mm, a parameter that was exceeded in part of El Khababi et al and Yuan et al datasets. The use of spasmolytics and enema is not a consensus [27, 28]. Three of the studies employed rectal microenema, while the other study did not provide this information. Two authors justified the use of microenema to reduce DWI susceptibility artifacts [9, 11]. Although it is not a consensus, this approach is aligned with evidence provided by a small sample single-center study that reported better quality in DWI with reduced geometric distortion from rectal gas [29]. Only Santiago et al explicitly reported the use of spasmolytics. The SAR and ESGAR consensus guidelines did not incorporate SSS or address other protocol-specific steps (e.g., the integrity of the hypointense mucosa layer could possibly be more conspicuously depicted with microenema).

Although the heterogeneity was high in our primary analysis, this finding was mostly associated with El Khababi et al study’s results, given the almost perfect threshold effect in our secondary analysis. This assumption was based on its outlier sensitivity, position in the SROC curve, and a significantly low IOA compared to the other studies. Hypothetical reasons in the study by El Khababi et al might be related to a higher number of readers (22 radiologists) and variable familiarity with the sign. Moreover, it has been hypothesized that the SSS might be very influenceable depending on T2WI acquisition quality, and part of the dataset was reported to be older and with non-standardized protocols, potentially increasing heterogeneity. However, heterogeneity evaluation should be interpreted with caution given the limited number of studies included in the analysis.

This study had limitations. First, there were only four studies that met the eligibility criteria, which might be related to the recent adoption of the term SSS in 2020 by Santiago et al [3]. Even though descriptions similar to the SSS such as “near-normalization of the wall and regular with a thin hypointense luminal scar” might have been previously used in the literature, the first structured report of the sign to predict complete response with standard reference was done in 2020. Second, it is important to consider possible biases from the primary studies, such as the retrospective nature of some of them, in which a memory bias cannot be excluded even with readers’ blinding. The difference in the percentage of patients undergoing surgery and clinical follow-up to establish the reference standard for recurrence is a potential source of selection bias. However, complete pathological response was the ground truth for 88.8% of cases in this meta-analysis, which limits some of the potential bias. Another limitation was the variable follow-up time in these studies, with some cases having follow-up periods shorter than 12 months, while a local relapse generally occurs in the first 18 to 24 months [30]. Our primary analysis showed significant heterogeneity, which was mostly related to the outlier performance of El Khababi et al as demonstrated in the secondary analysis. However, a more thorough analysis of heterogeneity and potential outliers is also significantly limited given the number of included studies.

In summary, the SSS has shown high specificity with variable sensitivity for complete response after neoadjuvant treatment. This imaging finding on rectal MRI can improve diagnostic value to rectal cancer treatment response evaluation and select candidates for a WW approach. This meta-analysis encourages further research with larger samples, multi-center approach, prospective design, and longer follow-up durations.

### Supplementary information

Below is the link to the electronic supplementary material.

Supplementary file1 (DOCX 479 KB)

## Abbreviations

AUC: Area under the curve
CRT: Chemoradiotherapy
DOR: Diagnostic odds ratio
DWI: Diffusion-weighted imaging
IOA: Interobserver agreement
LARC: Locally advanced rectal cancer
MRI: Magnetic resonance imaging
mrTRG: Magnetic resonance treatment regression grade
pCR: Pathological complete response
PICOS: Population, intervention, comparator, outcome, study design
PRISMA: Preferred Reporting Items for Systematic Reviews
PROSPERO: Prospective Register of Systematic Reviews Database
QUADAS: Quality Assessment of Diagnostic Accuracy Studies
SROC: Summary receiver-operating characteristic curve
SSS: Split scar sign
T2WI: T2-Weighted image
TME: Total mesorectal excision
WW: Watch-and-wait

## Declarations

### Guarantor

The scientific guarantor of this publication is Stephan Altmayer, MD, PhD.

### Statistics and biometry

Some of the authors have significant statistical expertise.

### Study subjects or cohorts overlap

The is no cohort overlap.

### Methodology

• retrospective

• systematic review and meta-analysis
