# Correction to: Split scar sign to predict complete response in rectal cancer after neoadjuvant chemoradiotherapy: systematic review and meta-analysis

**DOI:** 10.1007/s00330-023-10576-5

**Published:** 2024-01-19

**Authors:** Giovanni Brondani Torri, Camila Piovesan Wiethan, Felipe Welter Langer, Guilherme Strieder de Oliveira, Alice Villa Bella Meirelles, Natally Horvat, Justin Ruey Tse, Adriano Basso Dias, Stephan Altmayer

**Affiliations:** 1 Department of Radiology and Diagnostic Imaging, University Hospital of Santa Maria, Federal University of Santa Maria, Santa Maria, Rio Grande Do Sul 97105-900 Brazil; 2 Clinics Hospital of Porto Alegre, R. Ramiro Barcelos, Porto Alegre, 235090035903 Brazil; 3 Clinics Hospital, Federal University of Minas Gerais, Av. Prof. Alfredo Balena, Santa Efigênia, Belo Horizonte, 11030130-100 Brazil; 4 Department of Radiology, Memorial Sloan Kettering Cancer Center, 1275 York Ave, New York, NY 10065 USA; 5 Department of Radiology, Stanford University School of Medicine, 300 Pasteur Drive, Stanford, CA 94305-5105 USA; 6 Joint Department of Medical Imaging, University Medical Imaging Toronto, University Health Network–Mount Sinai Hospital–Women’s College Hospital, University of Toronto, 585 University Avenue, Toronto, Toronto, ON M5G 2N2 Canada

**Correction to: European Radiology**

10.1007/s00330-023-10447-z

In the original version of this article, the given and family names of Adriano Basso Dias (AB Dias) were incorrectly structured as Adriano Dias Basso (AD Basso).

The original article has been corrected.

