# Supplementary material for: Split scar sign to predict complete response in rectal cancer after neoadjuvant chemoradiotherapy: systematic review and meta-analysis
Source: Eur Radiol. 2023 Nov 18;34(6):3874–81. doi: 10.1007/s00330-023-10447-z (PMC11166750; doi:10.1007/s00330-023-10447-z)

## **Split Scar Sign to Predict Complete Response in Rectal Cancer after Neoadjuvant Chemoradiotherapy: Systematic Review and Meta-Analysis**

## **ELECTRONIC SUPPLEMENTARY MATERIAL**

## **Appendix E1: Search Strategy**

**(**(("magnetic resonance imaging" OR "MRI" OR "mr") OR ("split scar sign" OR "split scar" OR mrsss)) AND (response OR restaging OR re-staging) AND ("Colorectal Neoplasms" OR "Colorectal Cancer" OR "Colorectal Cancers" OR "Colorectal Carcinoma" OR "Colorectal Carcinomas" OR "Colorectal Neoplasm" OR "Colorectal Tumor" OR "Colorectal Tumors" OR "colorectal tumorigenesis" OR "colorectal tumour" OR "Colonic Neoplasms" OR "Cancer of Colon" OR "Cancer of the Colon" OR "Colon Adenocarcinoma" OR "Colon Cancer" OR "Colon Cancers" OR "Colon Neoplasm" OR "Colon Neoplasms" OR "Colonic Cancer" OR "Colonic Cancers" OR "Colonic Neoplasm" OR "Rectal Neoplasms" OR "Cancer of Rectum" OR "Cancer of the Rectum" OR "Rectal Cancer" OR "Rectal Cancers" OR "Rectal Neoplasm" OR "Rectal Tumor" OR "Rectal Tumors" OR "Rectum Cancer" OR "Rectum Cancers" OR "Rectum Neoplasm" OR "Rectum Neoplasms")

**Results have been filtered to exclude articles before 2020.**

## **Table E1:** Studies’ characteristics.

|  | **Santiago 2020** | **Popita 2022** | **El Khababi 2022** | **Yuan 2023** |
| --- | --- | --- | --- | --- |
| **Sample size** | 58 | 40 | 90 | 189 |
| **Age** | < 60 years: 29 (50%)  > 60 years: 29 (50%) | < 49: 10 (25%)  50-59: 9 (22.5%)  60-69: 11 (27.5%)  > 70: 10 (25%) | Mean 65 years (+-11) | Median: 58 years (50-67) |
| **Male** | 33 (57%) | M: 23 (57.5%) | M: 52 (58%) | M: 131 (69.3%) |
| **Initial T staging** | < T3b: 38 (69%) O1; 34 (62%) O2  > T3c: 17 (31%) O1; 21 (38%) O2 | N/A | cT1-2: 3 (3%)  cT3: 68 (76%)  cT4: 18 (20%) | cT2: 15 (7.9%)  cT3: 156 (82.5%)  cT4: 18 (9.5%) |
| **Distance from anal verge** | < 6 cm: 27 (49%) O1; 23 (42%) O2  > 6 cm: 28 (51%) O1; 32 (58%) O2 | < 6 cm: 18 (45%)  > 6cm: 22 (55%) | N/A | N/A |
| **Initial N staging** | N+: 23 (42%) O1; 35 (64%) O2  N0: 32 (58%) O1; 20 (36%) O2 | N/A | cN0: 12 (13%)  cN1: 19 (21%)  cN2: 59 (66%) | cN0: 35 (18.5%)  cN+: 154 (81.5%) |
| **Mean interval after radiotherapy** | 9.1 weeks | 6.5 weeks | N/A | N/A |
| **MEASUREMENTS AND OUTCOMES** |  |  |  |  |
| **Number of observers** | 2 | 2 | 22 | 3 |
| **Number of experts** | 2 | 2 | 5 | N/A |
| **Reader time experience** | 10-13 years | 8-9 years | >10 years | N/A |
| **Imaging Technique** | 1,5 T - Performed after enema and IV butiylscopolamine | 1,5 T - Performed after enema | N/A | 3,0 T - Performed after enema |
| **Reference Standard** | pTRG or Clinical follow-up (Minimun 1 year) | pTRG, Endoscopy or Clinical follow-up (Minimun 6 months) | pTRG or Clinical follow-up (Minimun 2 years) | pTRG |
| **Complete response / Sample** | 25/58 = 43% | 21/40 = 52.5% | 27/90 = 30% | 41/189 = 21.7% |
| **Average IOA** | 0.69 | 0.8 | 0.17 | 0.899 |

##

## **Table E2:** MRI Protocols

| **Author** | **Vendor** | **Sequence** | **TE (ms)** | **TR**  **(ms)** | **ETL** | **Slice thickness (mm)** | **Gap** | **Matrix** | **FOV (mm or mm²)** | **b-values** |
| --- | --- | --- | --- | --- | --- | --- | --- | --- | --- | --- |
| **Santiago et al.** | 1.5 T Achieva /1.5 T Ingenia Philips (A/I) | Axial T2WI | 120/85 | 3000/5692 | 21/18 | 3/3 | 0.5/0.3 | 248×242 / 416×46 | 200×200 / 250×328 | - |
|  |  | Axial DWI | 69/90 | 2000/4288 | - | 3/5 | 1/0 | 124×124 / 76×65 | 370×370 / 200×200 | 0, 200, 500, 1000 / 0, 1400 |
| **Popita et al.** | 1.5 T Magnetom Aera Siemens | Axial T2WI | 114 | 6380 | 17 | 3 | 0.9 | 166×384 | 360 mm² | - |
|  |  | Axial DWI | 75 | 6700 | - | 3 | 0 | 126 ×126 | 220 mm² | 50, 500, 1000, 1500 |
| **El Khababi et al.** | Non-standardized | Axial T2WI | N/A | N/A | N/A | 3-5 | N/A | N/A | N/A | - |
|  |  | Axial DWI | N/A | N/A | N/A | N/A | N/A | N/A | N/A | high b between 600 - 1200 |
| **Yuan et al.** | 3.0 T Discovery 750w GE / 3.0 T Magnetom Skyra Siemens (D/M) | Axial T2WI | 116/108 | 6538/4000 | 32/16 | 4/3 | N/A | 352×352 / 320×320 | 200×200 /  180×180 | - |
|  |  | Axial DWI | Minimum/89 | 2840/6300 | - | 6/5 | N/A | 128×128 / 150×150 | 320×256 / 380×380 | 0, 1000/0, 1000 |

A/I: 1.5 T Achieva /1.5 T Ingenia Philips

D/M: 3.0 T Discovery 750w GE / 3.0 T Magnetom Skyra Siemens

TE: echo time

TE: repetition time

ETL: echo train length

FOV: field of view

## **Figures E1:** Study Quality Assessment According QUADAS-2


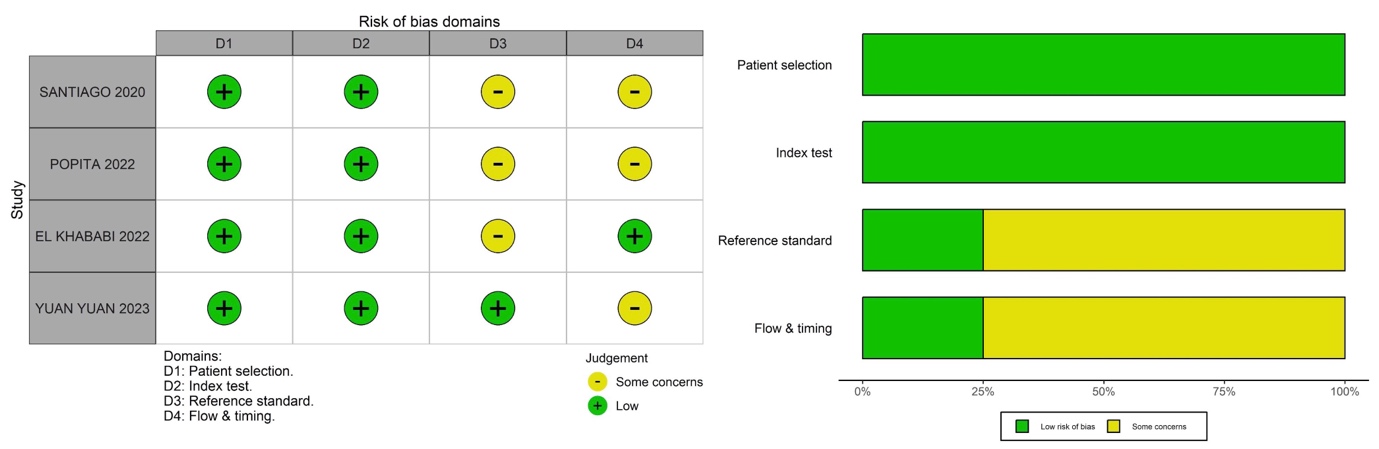


## **Figures E2:** Forest plot after removing El Khababi et al.


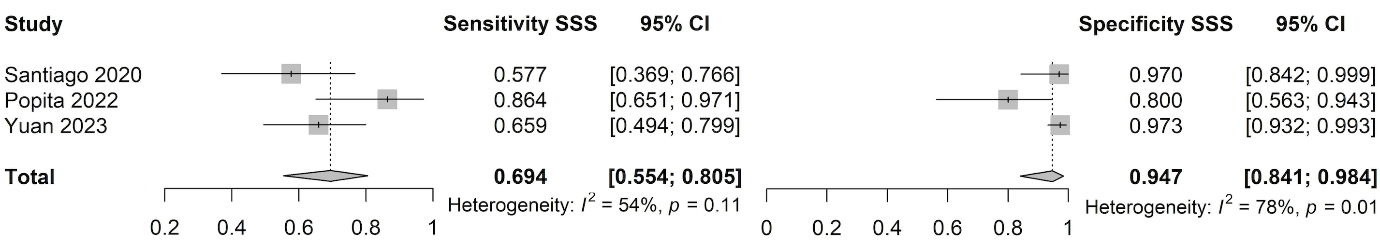


## **Figures E3:** Forest plot after removing El Khababi et al.


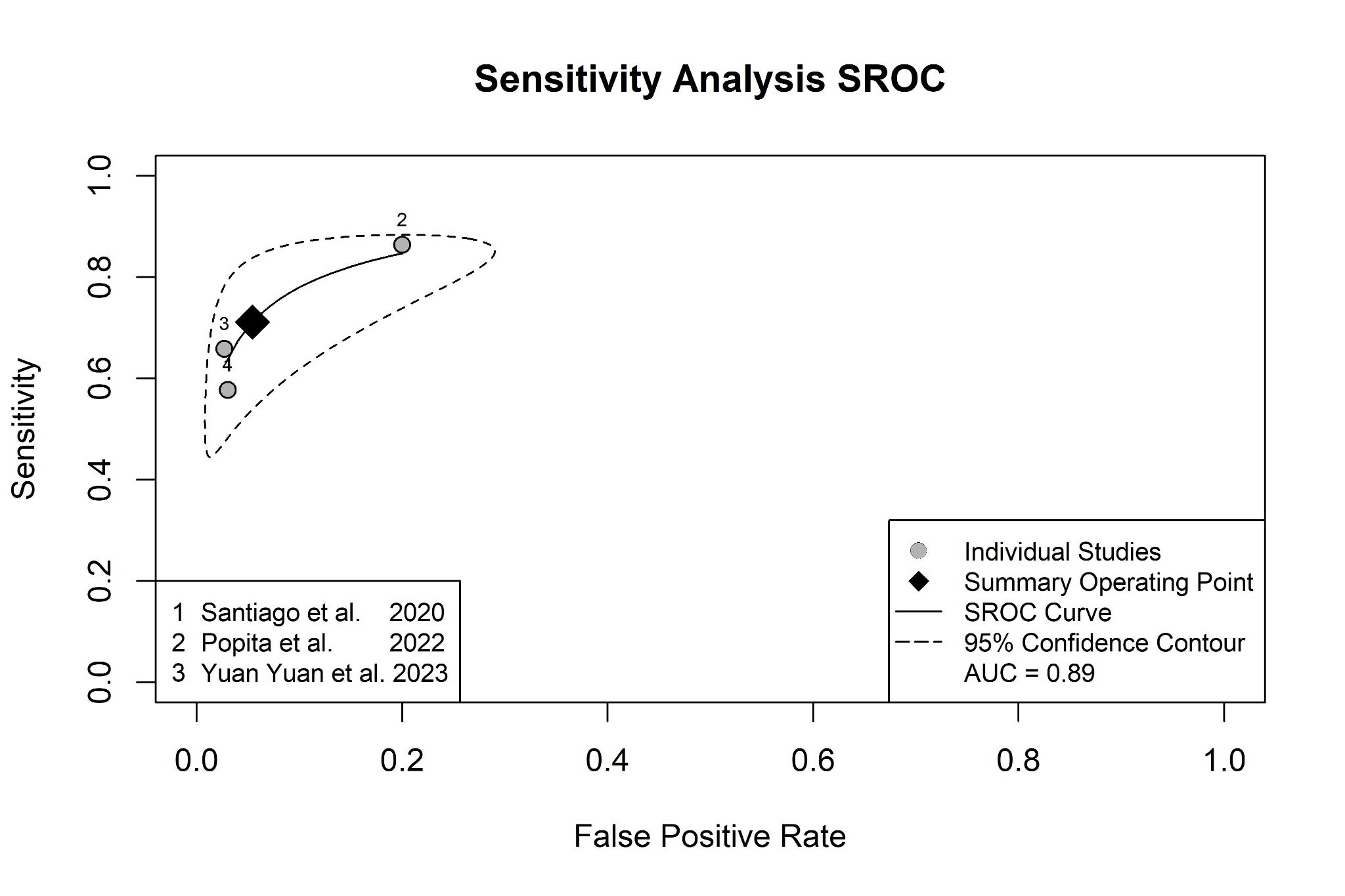

Supplement: Supplementary file 1 — Supplementary file1 (DOCX 479 KB) [file 330_2023_10447_MOESM1_ESM.docx]
